# Case series report: use of vibroacoustic pulmonary therapy in patients with thoracic trauma complicated by acute respiratory failure

**DOI:** 10.3389/fmed.2024.1399397

**Published:** 2024-09-04

**Authors:** Aidos Konkayev, Assema Bekniyazova, Zaituna Khamidullina, Maiya Konkayeva

**Affiliations:** ^1^Astana Medical University, Astana, Kazakhstan; ^2^The National Scientific Center of Traumatology and Orthopedics named after Academician Batpenov N.D., Astana, Kazakhstan; ^3^National Research Oncology Centre, Astana, Kazakhstan

**Keywords:** respiratory failure, polytrauma, vibroacoustic therapy, chest trauma, case series

## Abstract

Chest injury is often accompanied by polytrauma and is complicated by respiratory failure. This article presents a series of cases with verified acute respiratory failure in patients with chest injury, where vibroacoustic pulmonary therapy was used in complex treatment. Dynamic X-rays and respiratory drive indicators reflected the effect of the use of vibroacoustic lung therapy. Early diagnosis of respiratory disorders and complex therapy using vibroacoustic pulmonary therapy can improve results. As a result, the time spent in the hospital and in the intensive care unit is reduced, and the frequency of adverse outcomes is reduced.

## Introduction

Chest trauma accompanies polytrauma in almost half of cases. It also triggers an inflammatory reaction, which ultimately leads to respiratory disorders (1). To prevent the occurrence of respiratory complications, such as pneumonia and acute respiratory distress syndrome (ARDS), the time of artificial ventilation should be kept to a minimum (2). Age, number of ribs involved, and development of pneumonia, pulmonary contusions, and bilateral chest wall involvement may lead to increased morbidity and mortality in chest trauma (3).

The resulting chest injury can become a source of comorbidity and requires the prevention of serious pulmonary complications in this category of patients through rational treatment in combination with a good physiotherapeutic approach (4).

Thoracic trauma as a component of polytrauma is the main factor in the death of patients in up to 60% of cases. Both chest trauma and lung contusions can cause respiratory distress. This is demonstrated by the published association between mortality and Injury Severity Score (ISS) >25 after severe chest trauma. In this regard, a comprehensive approach and targeted pulmonary rehabilitation is important and can improve patient outcomes (5).

With initial cardiopulmonary pathology, the risk of an unfavorable outcome increases progressively. The development of pneumonia after injury increases the risk of complications (6). But with a timely start, negative outcomes are reversible, even in the absence of signs in the initial phase of inflammation (7).

The presented article describes a series of patients with existing chest trauma complicated by respiratory failure. As necessary, blood sampling for tests, chest X-ray, ultrasound, video bronchoscopy, bacteriological discharge for targeted selection of antibiotic therapy, and rehabilitation measures were carried out (Tables 1–3).

**Table 1:** General information about patients.

Clinical case	1	2	3
Date of admission to the hospital	08/28/23	07/13/23	08/10/23
Age	49	26	44
BMI	29	32	33
Sex	M	М	W
Race	Asian	Asian	Caucasian
Allergoanamnesis	No	No	No
Heredity	Not burdened	Not burdened	Not burdened
Social status	Disabled, not working	Worker	Worker
The cause of the injury	Fall from a height of 10 meters	Road accident (moped driver)	Road accident (passenger)
Diagnosis	Combined injury. Closed craniocerebral injury. Moderate brain contusion, acute period. Subarachnoid hemorrhage. Closed unstable uncomplicated compression-comminuted fracture of the bodies of Th11, L2, L3 vertebrae, 2nd degree. Closed stable uncomplicated compression fracture of the Th6 vertebral body, 1st degree. Fracture of the right and left transverse processes of L2, L3, L4, L5 vertebrae, right transverse processes of Th10, Th12, L1 vertebrae. Blunt chest trauma. Contusion of both lungs. Closed fracture of ribs 2, 3, 4, 7 on the left, without displacement, ribs 5, 6 on the left, with displacement of fragments. Fracture of the 5th and 6th ribs on the right with displacement of fragments, 6th ribs on the right without displacement. Blunt abdominal trauma. Retroperitoneal hematoma. Closed comminuted fracture of the right and left lateral masses of the sacrum, body with complete dislocation of the S2 vertebra, with displacement of bone fragments. Fracture of the body of the right pubic and ischial bone with displacement of bone fragments. Closed fracture of the anterior and posterior columns, the bottom of the acetabulum on the right with displacement of bone fragments. Open II B according to Kaplan—Markova intra-articular comminuted fracture of the right calcaneus with displacement of bone fragments. Open II B according to Kaplan—Markova intra-articular comminuted fracture of the right talus with displacement of bone fragments. Closed comminuted fracture of the distal metaepiphysis of both bones of the right leg with displacement of bone fragments. Open I A according to Kaplan—Markova comminuted intra-articular fracture of the left calcaneus with displacement of bone fragments. Open I A according to Kaplan—Markova fracture-dislocation of the left talus with displacement of bone fragments. Bruises, abrasions of the soft tissues of the face. Traumatic shock of the 3rd degree. Hemorrhagic shock of the 3rd degree	Combined injury. Closed craniocerebral injury. Brain concussion. Acute period. Closed comminuted fracture of the middle third of the right clavicle with displacement of bone fragments. Closed fracture of the acromial end of the left clavicle without displacement of bone fragments. Closed fracture of the manubrium and body of the sternum without displacement of bone fragments. Closed fracture of both pubic bones, lateral mass of the sacrum on the left with displacement of bone fragments. Closed fracture of ribs 1–7 on the left, 1–7 on the right. Contusion of the chest organs, mediastinum. Bruises of both lungs. Pneumothorax on both sides. Bruised wound on the chin. Bruises, abrasions, subcutaneous hematomas of the scalp and face. Bruised wounds in the area of the lower third of the left shoulder and elbow joint. Bruise, abrasions of both knee joints. Abrasions on both hands and right foot	Blunt chest trauma. Fracture of 7–12 ribs on the left, 5–11 ribs on the right without displacement. Bilateral hemothorax. Fracture of the sternum. Hemomediastinum. Bilateral pelvic fracture. Hemorrhagic shock 4 degrees
Concomitant pathology	Schizophrenia	No	No
ISS	57	45	50

**Table 2:** Information about the disease.

Case number	1	2	3
Ventilation upon admission	Yes	Yes	Yes
Length of stay in ICU and hospital	38 out of 46 days	26 out of 41 days	12 out of 19 days
Surgery	1. Application of a device for compression-distraction osteosynthesis (08/28/23) 2. Tracheostomy (09/05/23) 3. Closed reduction of bone fragments of the tarsal and metatarsal bones with internal fixation (07/09/23)	1. Thoractomy, osteosynthesis of ribs 2. Tracheostomy (07/26/23)	1. Thoractomy, osteosynthesis of ribs
Pneumonia diagnosis day	09/07/23	07/20/23	08/14/23
Bacteriological culture of alveolar lavage	*Acinetobacter baumannii* 3*10^5^	*Acinetobacter baumannii* 3*10^2^	*Acinetobacter baumannii* 3*10^3^
Mechanical ventilation, days	08/28/23–10/02/23	07/13/23–07/30/23	08/14/23–08/19/23
Pneumonia Resolution Day	09/27/23	08/13/23	08/21/23
Lung compliance before and after VALT	37 61	42 76	45 74
P/F Before and after VALT mmHg	179–213	206–250	189–300

**Table 3:** Therapy.

Antibiotic	Ceftriaxone (2 g IV once a day) (08/28/23–09/07/23) Levofloxacin (500 mg Intravenously) 2 times a day (09/07/23–09/20/23) Amikacin (500 mg Intravenously) 3 times a day (09/13/23–09/20/23) Cefepime (1 g Intravenously) 2 times a day (09/20/23–10/13/23)	Ceftriaxone (2 g IV once a day) (07/13/23–07/17/23) + Levofloxacin 500 mg Intravenously 2 times a day Meropenem (1,000 mg IV tid.) Vancomycin 1 g Intravenous micro-jet 3 times a day	Ceftriaxone (2 g IV once a day) (08/10/23–18/10/23) + Levofloxacin 500 mg Intravenously 2 times a day Meropenem (1,000 mg IV tid.) Vancomycin 1 g Intravenous micro-jet 3 times a day
Gastroprotector	Omeprazole 20 mg by tube 1 time per day	Omeprazole 20 mg by tube 1 time per day	Omeprazole 20 mg by tube 1 time per day
Anticoagulant	–	–	–
Disaggregant	Acetylsalicylic acid (100 mg 1 time per day, by tube)	Acetylsalicylic acid (100 mg 1 time per day, by tube)	Acetylsalicylic acid (100 mg 1 time per day, by tube)
Diuretic	20 mg Intravenously [1 time per day (periodically if necessary)]	20 mg Intravenously [1 time per day (periodically if necessary)]	20 mg Intravenously [1 time per day (periodically if necessary)]
Steroid	–	–	–
Non-steroidal (amount varies according to need)	Analgin (1,000 mg Intravenous) Ketoprofen (100 mg, i.v.)	Analgin (1,000 mg Intravenous) Ketoprofen (100 mg, i.v.)	Analgin (1,000 mg Intravenous) Ketoprofen (100 mg, i.v.)
Opioids (quantity varies according to need)	Promedol (20 mg intravenously) Morphine hydrochloride (10 mg intravenously)	Promedol (20 mg intravenously) Morphine hydrochloride (10 mg intravenously)	Promedol (20 mg intravenously)
Sedation	Dexdor (200 mcg/Intravenous (perfusor)) (1 time per day)	Dexdor (200 mcg/Intravenous (perfusor)) (1 time per day)	Dexdor (200 mcg/Intravenous (perfusor)) (1 time per day)
Antihistamines	Diphenhydramine (10 mg IV twice a day)	Diphenhydramine (10 mg IV twice a day)	–
Mucolytics	Ambroxol hydrochloride (15 m Inhalation 4 times a day)	Ambroxol hydrochloride (15 m Inhalation 4 times a day)	Ambroxol hydrochloride (15 m Inhalation 4 times a day)
Infusion-transfusion therapy	Saline solutions Fresh frozen plasma, washed red blood cells, 10% albumin	Saline solutions Fresh frozen plasma, washed red blood cells, 10% albumin	Saline solutions Fresh frozen plasma, washed red blood cells, 10% albumin
Other drugs	Tizercin (25 mg Orally) 1 time per day Azaleptol (25 mg, Tablets) (25 mg by tube) (1 r/d) Adrenaline (3.6 mg Intravenously through a perfuser); Norepinephrine Agetan (8 mg Intravenously through a perfuser) Dopamine (400 mg Intravenously through a perfuser)	Adrenaline (3.6 mg Intravenously through a perfuser); Norepinephrine Agetan (8 mg Intravenously through a perfuser) Dopamine (400 mg Intravenously through a perfuser)	Adrenaline (3.6 mg Intravenously through a perfuser); Norepinephrine Agetan (8 mg Intravenously through a perfuser) Dopamine (400 mg Intravenously through a perfuser)
Parenteral nutrition	25–30 kcal/kg/day	25–30 kcal/kg/day	25–30 kcal/kg/day

*Volemic status was assessed by measuring the central venous pressure of patients and, if necessary, stimulated with intravenous furosemide 20 mg 2 times a day. If necessary, up to 100 mg of the drug was administered using a dispenser during the day under the control of central venous pressure.

All patients underwent correction of metabolic and electrolyte disturbances at different times as needed. Patients were prescribed sedatives, analgesics, antiarrhythmics, antihypertensive drugs, and adrenergic agonists according to indications under the control of hemodynamic parameters.

Vibroacoustic therapy of the lungs (VALT) was connected immediately after verification of respiratory failure. Vibroacoustic therapy (VAT) combines acoustic and vibration effects with intense waves and creates mechanical vibrations. The main mechanism of the therapeutic effect of VALT is the use of sound waves with frequencies from 20 to 300 Hz, which create a resonance effect in the patient’s body, coinciding with the resonance frequencies of the chest, lung tissue and bronchi (8).

Thus, further research is needed to confirm the effectiveness of vibroacoustic therapy, which may have had an important role in the treatment of respiratory distress in patients with chest trauma.

### Patient information

All patients were admitted to the ward called “anti-shock,” which is located near the entrance to the hospital and is a key unit where the patient is delivered, bypassing the admission department, beforehand the ambulance service notifies the hospital staff to provide immediate assistance. As part of the multidisciplinary duty team, medical examinations are carried out, central vein catheterization, if necessary—tracheal intubation and artificial ventilation, laboratory and instrumental diagnostics and, if necessary, emergency surgical interventions are carried out, as well as monitoring of functional indicators in the process of stopping traumatic shock.

#### First case

A 49-year-old patient was admitted in the morning, 1 h after the injury. The injury was sustained as a result of a fall from a height of 10 meters “feet down” (Table 1). The allergy history is normal. The hereditary history is not burdened. The patient is dynamically monitored by a psychiatrist with a diagnosis of “manic schizophrenia,” periodically receives inpatient treatment in a specialized department and receives antipsychotic drugs.

The condition upon admission is extremely serious. The assessment on the Injury Severity Score (ISS) is 57 points. The patient was examined by specialized specialists, X-ray, ultrasound, CT (computer tomography), ECG were performed, blood tests were taken and he was urgently hospitalized in the intensive care unit. After relative stabilization of the patient’s condition in the anti-shock ward, he was transferred to the intensive care unit for further treatment and transported on an artificial lung ventilation apparatus.

In the evening of the same day, an operation was performed in the volume—application of the Hoffman external fixation device on the pelvic bones, the right lower leg with the capture of the ankle joint and the foot on both sides.

On the 7th day, a tracheostomy was applied, on the same day, a closed reposition of the distal meta epiphysis of the right tibia, both calcaneus bones, closed synthesis with pins was performed.

On the 8th day, the patient has a subfebrile temperature with chills. The patient was further examined, an X-ray was performed, left-sided pneumonia was detected. Bacterial culture from bronchoalveolar lavage—*Acinetobacter baumannii* 3 * 10^5^. The patient was on artificial ventilation from the moment of admission for 34 days. The patient’s respiratory parameters were assessed during the observation period (Table 2). The patient’s treatment is described in Table 3.

**Figure 1:**
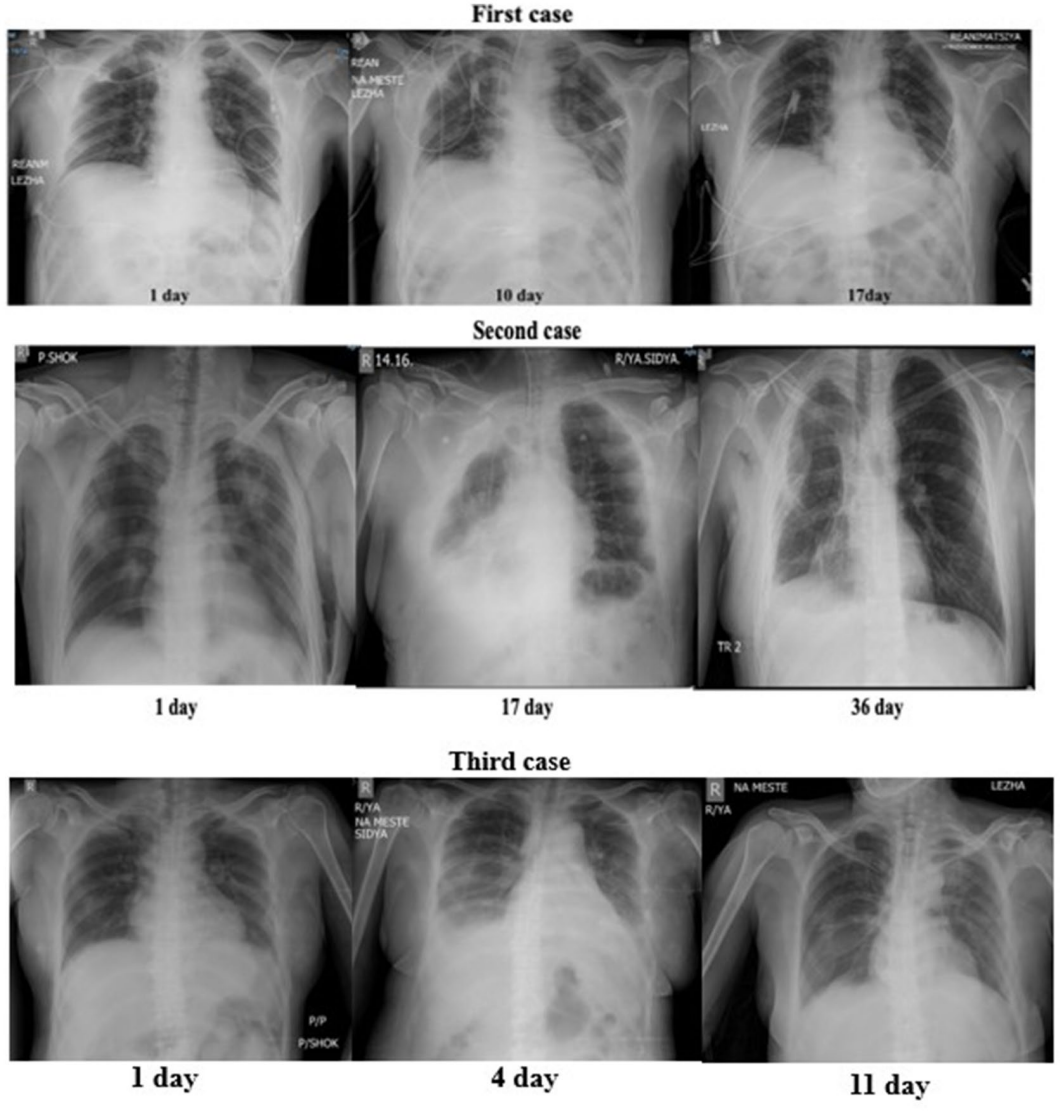
X-ray dynamic.

On the 29th day after the regression of pneumonia complicated by respiratory failure (Table 2), a test for independent breathing was performed, after which the patient was successfully transferred to independent breathing (Figure 1). On the 38th day, he was transferred to a specialized department. On the 46th day, he was discharged home in a satisfactory condition.

Patient was re-admitted to the rehabilitation department a month later, given the absence of complaints from the respiratory system, no examination was performed.

#### Second case

A 26-year-old patient was admitted with an injury as a result of a traffic accident (moped driver) (Table 1). Allergy history and heredity are unremarkable. Life history is unremarkable. The condition upon admission is extremely serious due to shock and blood loss. ISS 45 points. The patient was examined by specialized specialists, X-ray, ultrasound, CT, ECG were performed, blood tests were taken and the patient was urgently hospitalized in the intensive care unit. From the moment of admission, he was on artificial ventilation. On the 8th day, thoracotomy and rib osteosynthesis were performed. On the 13th day, a tracheostomy was installed.

On the 17th day after admission, the patient’s CT scan revealed signs of pleuropneumonia of the right lung with the presence of a cystic formation in the apex of the lung (a developing abscess) (Table 2). The study of bronchoalveolar lavage revealed-*Acinetobacter baumannii* 3*10^2^.

The patient underwent VALT therapy after stabilization of the chest frame, since sharply displaced rib fractures are a contraindication for sessions.

In total, the patient was in the hospital for 41 days, of which 26 days were in the intensive care unit, 17 of which were on mechanical ventilation. Resolution of pneumonia was on the 30th day of hospital stay.

During the observation period in the intensive care unit, the patient’s respiratory parameters were assessed (Table 2). The patient’s treatment is described in Table 3.

The patient was re-admitted to the rehabilitation department 10 months after discharge from the hospital due to an incorrectly healed fracture of the right clavicle. Post-traumatic injury to the long thoracic nerve on the right. On the computed tomography of the chest organs of this patient, the following is noted: signs of pneumofibrosis of the upper and middle lobes of the right lung.

#### Third case

A 44-year-old patient was admitted to the anti-shock department as a result of a car accident (Table 1). The medical history is unremarkable. ISS 50 points. The patient was consulted by specialized specialists and laboratory and instrumental studies were performed. After relative stabilization of the condition, he was transferred to the intensive care unit on a portable artificial lung ventilation device.

The patient was on artificial lung ventilation for 2 days after admission, after which he was extubated and again transferred to artificial lung ventilation due to complications of respiratory failure in the form of pneumonia (Table 2). Bacterial culture from bracheoalveolar lavage—*Acinetobacter baumannii* 3 * 10^3^. He was in the hospital for 19 days, including 12 in the intensive care unit, of which 5 days he was on artificial lung ventilation. Respiratory system parameters and treatment are listed in Tables 2, 3. Тhere is no data on rehabilitation and further medical history, since the patient is a citizen of another country.

## Methods

All patients signed informed consent, and the project was approved by the Research Ethics Committee of the National Scientific Center of Traumatology and Orthopedics named after Academician N.D. Batpenov on January 2, 2022. The study was conducted in accordance with the protocol of the Ministry of Health of the Republic of Kazakhstan dated September 16, 2022 Protocol No. 169.

*Inclusion criteria:*

- chest trauma;
- age over 18 years;
- respiratory failure.

*Exclusion criteria:*

- terminal condition of the patient;
- shock;
- paradoxical pathological breathing;
- hypertensive crisis;
- severe hypocoagulation with the risk of hematoma or bleeding in the projection of exposure;
- severe hypercoagulation, risk of thrombus/embolus migration along the main vessels in the projection of exposure;
- acute cerebrovascular accident (CVA) in the first 1–3 days;
- cerebral edema;
- the presence of multiple purulent or burn wound surfaces in the projection of exposure;
- the presence of unstable comminuted rib fracture;
- osteomyelitis of the ribs and/or thoracic spine; spinal fracture without orthopedic fixation;
- chest or abdominal trauma with bleeding.

Vibroacoustic therapy is started after rib osteosynthesis and stabilization of the patient’s critical condition. The emitters of the device were applied to the most affected areas of the patient’s lungs, corresponding to the CT or radiography data, and one or the other mode of the device was turned on. Because the device has long cords for emitters and is portable, the patient’s position does not matter and does not require active participation, which is important for patients undergoing artificial lung ventilation. Immediately before the procedure, arterial blood was collected once in the morning to determine PaO2, PaCO2, and P/F blood, as well as 10 min after the session.

The effectiveness of the impact increases with the increase in the frequency, but not with the increase in the duration of the procedure (session). The effective duration of the procedure is 3–5 min. The device has a power of impact on the chest from 0 to 100%, in these patients a weak power of 20–30% was used to prevent rib retraumatization. The procedure was carried out every 6–8 sessions every 2 h mainly in the daytime using a vibroacoustic device. A short-term decrease in saturation up to 30 s was noted, associated with active sputum discharge. Clinically, when using the device, an improvement in the drainage function of the bronchi was observed. The drainage effect was also recorded visually during bronchoscopy.

All patients after osteosynthesis and stabilization of the critical condition underwent rehabilitation measures to prevent cardiorespiratory complications and deep vein thrombosis. In this case, fast-track rehabilitation and enhanced recovery after surgery (ERAS) were used. Early initiation of rehabilitation treatment, on days 2–5, was started and aimed at restoring the range of motion in the operated part of the body, restoring tone and increasing muscle strength, preventing cardiorespiratory disorders in order to improve functional results after surgery in thoracic surgery. After consciousness was restored, the stage of activating patients within the bed followed. Then, after extubation and decannulation of patients, breathing exercises, low-intensity therapeutic physical training, passive kinesitherapy, activating the patient outside the bed, and therapeutic massage.

### Follow-up and outcomes

The effectiveness of the physiotherapy component was assessed by X-ray (Figure 1) and indices of the oxygenation index and lung compliance of patients before and after the use of VALT with standard treatment according to the protocol for the management of patients with respiratory insufficiency (Tables 2, 3).

The patients had no contraindications to vibroacoustic pulmonary therapy, as well as undesirable consequences after the procedure. A tolerance test was performed using a short-term use of a device for vibroacoustic lung therapy lasting up to 1 min with an assessment of hemodynamics, saturation and sensitivity of the patient.

## Discussion

The strengths of this study are that patients with severe injuries and a high risk of adverse outcomes had a favorable outcome, transferred to specialized departments, then home, which is confirmed by the admission of two patients objectively upon re-hospitalization to the rehabilitation department.

Limitations are a series of cases with a small sample, where statistical analysis of the data is not representative. Taking this into account, a prospective study of a group using a vibroacoustic device and a control group without it in this category of patients is planned in the future.

In general, a multidisciplinary and integrated approach improves patient outcomes (9). Vibroacoustic therapy of the lungs affects blood flow by improving the perfusion of pathological lung tissue (10). Sessions were conducted using the VibroLung apparatus, specially designed for vibroacoustic “massage” of the chest (11).

In medicine, vibroacoustic therapy began at the end of the 20th century, but to this day there is not enough information in evidence-based medicine databases (12).

In a study on pain treatment, vibroacoustic therapy with a frequency of 40 Hz, the duration of sessions varied from 20 to 45 min. The frequency of therapy was higher for the treatment of acute pain compared to chronic pain (13).

Also in the treatment of children with cerebral palsy, two studies used a frequency of 40 Hz, as recommended by Skille. Liu’s team decided to use a frequency of 60 Hz, and in another study Liu used 16–150 Hz. Wigram used a sine wave sound with a frequency of 44 Hz, which is close to the 40 Hz used by Katusik. In the study by Kvam, a frequency range of 40–80 Hz was used (14).

Studies in patients with Parkinson’s disease confirm that VAT is effective in reducing tremor using passive low-frequency sound vibrations in the range of 20–100 Hz (15). Also, a review of the literature devoted to the study of the mechanism of the impact of sound vibration on humans, covering physiological, neurological and biochemical mechanisms, reflects the analysis of music, moving to sound, and then to vibration, with special attention to low-frequency sound (up to 250 Hz) and infrasound (1–16 Hz). It should also be noted that in this study, at the level of the mechanism, whole-body vibration (WBV) and VAT are considered within the same area of pulse stimulation, since it has an intersection of the frequency range from 1 to 15 Hz (16).

Based on the manufacturer’s instructions, triangular modulated vibroacoustic waves with a relatively “slow” period (1.1–10 s) are used to improve the ventilation/perfusion relationship and to promote recruitment and drainage of large bronchi. “Faster” modulations (0.2–1.0 s) with varying frequencies are used to improve drainage of smaller bronchi and alveolar sacs. The acoustic characteristics of the chest wall cause the frequency of these waves to decrease rapidly, creating an effect similar to gentle tapping, while the rapidly increasing frequency simulates gentle pressure, promoting mucus clearance. Clinical observations indicate that frequencies between 20 and 60 Hz are most effective for treating the chest wall, with 37–42 Hz being particularly effective in creating optimal pressure oscillations in the large airways (11).

## Conclusion

There is a question regarding the effective frequency range, amplitude, duration and frequency of application of vibroacoustic therapy. The above-mentioned studies provide different data, so a study with a control group is of scientific interest.

## Patient’s point of view

According to patients, hardware massage turned out to be more pleasant than manual massage. In addition, vibroacoustic pulmonary therapy using the device contributed to greater cough relief and improved general condition and well-being of the patient.

## Author’s note

The authors have consulted the CARE Checklist (2013), and the manuscript was prepared and edited in accordance with the CARE Checklist (2013).

## Data Availability

The original contributions presented in the study are included in the article/Supplementary material, further inquiries can be directed to the corresponding author.
